# Association of Summer Heat Waves and the Probability of Preterm Birth in Minnesota: An Exploration of the Intersection of Race and Education

**DOI:** 10.3390/ijerph17176391

**Published:** 2020-09-02

**Authors:** M. Luke Smith, Rachel R. Hardeman

**Affiliations:** 1 Minnesota Population Center, University of Minnesota, Twin Cities, Minneapolis, MN 55455, USA; hard0222@umn.edu; 2 Division of Epidemiology and Community Health, University of Minnesota Twin Cities, Minneapolis, MN 55455, USA; 3 Division of Health Policy and Management, University of Minnesota Twin Cities, 420 Delaware St. SE, Minneapolis, MN 55455, USA; 4 Measuring & Operationalizing Racism to achieve Health Equity Lab, Minneapolis, MN 55455, USA

**Keywords:** maternal and child health, health equity, social determinants of health, climate change, heat waves, preterm birth, racism

## Abstract

Preterm birth (PTB) is common and has negative impacts on infant health. While some maternal risk factors have been identified, including age under 20 or over 40, substance abuse, low BMI, and racism, less is known about the impact of environmental exposures like high heat. We combined 154,157 records of live births occurring in Minnesota between 2009 and 2015 with hourly weather records collected from the Minneapolis–St. Paul airport. We tested if maternal heat wave exposure (a seven-day period with a mean daily high temp of 37 °C) immediately prior to birth leads to a higher risk of preterm birth. Additional covariates included maternal age, race/ethnicity, educational status, and residence in the seven-county Minneapolis–St. Paul metro area. Pregnant women exposed to a seven-day heat wave of 37 °C or higher experienced a higher relative risk of PTB compared to women who did not experience a heat wave (1.14 risk ratio (RR), 1.0–1.3 95% confidence interval (CI)). The result is robust to controls for a woman’s age, race/ethnicity, educational attainment, place of residence, and year of the birth. Children born to Black women with college degrees who are exposed to heat waves experience a higher relative risk of PTB compared to White women with college degrees in a heat wave (2.97 RR, 1.5–6.1 95% CI). Summer heat waves are associated with higher risk of PTB in late-term pregnancies in Minnesota.

## 1. Introduction

Preterm birth (PTB), or birth before 37 weeks of gestation, is a leading cause of infant mortality [1]. Approximately 10% of births in the United States are preterm [2], and 36% of infant deaths are attributable to PTB [1]. While improvements in survival of preterm infants have reduced the risk of death, preterm infants can have serious short- and long-term health issues including medical costs, lifelong developmental delays, and lower economic productivity [2]. From 2007 to 2014, PTB declined, largely due to reductions in teen pregnancy [3], but individual and societal effects of PTB in the United States remain high. Since 2014, PTB rates have again been trending upwards, suggesting that PTB will remain a significant societal challenge moving forward [4].

Well-documented risk factors of PTB include severely low maternal BMI, life stress, malnutrition, short intervals between pregnancies, substance abuse, hypertension, diabetes, some infections, and injury or trauma [5]. Historically disadvantaged populations like Black and Indigenous women also have higher rates of PTB [5,6,7]. Risk of PTB tends to be higher to children of individuals who are teens and over age 40 [8,9]. While education is protective in many health outcomes, higher education is not seen as protective against PTB to women who are Black [10], particularly when comparing PTB rate differences between children of college-educated women who are Black to births to college-educated women who are White [11,12]. The negative health outcomes and risk factors for PTB are well studied, but there are still gaps in the understanding of the etiology of PTB [13].

A number of studies have shown a link between heat waves and human harm in terms of all-cause mortality and morbidity [14,15]. Given changes in global climate, one potentially important but understudied risk factor for PTB may be high heat. There is growing research into the extent to which seasonality and environmental shocks such as heat waves may trigger PTB including systematic reviews by Olah et al. and by Zhang et al. [16,17] that show a general pattern of association between heat waves and PTB (along with still birth and low birth weight), but highlight a heterogeneity of measures and outcomes. Further studies showing a positive correlation between heat and PTB were conducted in China, Australia, Spain, the United States, Israel, and Italy [13,18,19,20,21,22,23,24,25,26,27,28]. Other studies, in the United States, Germany, Canada, and the United Kingdom, however, found no association [29,30,31,32] or even found a protective effect [33].

The understanding of the biological path from high heat to PTB is limited, but some research on animals finds positive correlations between heat waves and adverse physiological changes in litters born to sows (pigs) that include lower birth weight, PTB, and impaired development [34]. Other research finds an association between heat waves and changes to oxytocin and antidiuretic hormones which can lead to reduced uterine blood flow and less oxygen to lamb fetuses [35]. Finally, heat waves have been shown to be correlated with PTB and preterm rupture of membranes in humans [36]. While a variety of studies have explored the potential relationship between heat waves and PTB in humans, including Zhang et al.’s 2017 review of the literature [17], this is far from settled science as exposure definitions, study methodologies, and study outcomes are varied. As climatological models predict higher overall temperatures and greater severity and frequency of heat events both globally and within the United States, further research is warranted [37,38].

## 2. Materials and Methods

### 2.1. Outcome and Exposure

We conducted a retrospective study of all live summer births (defined as June 1–September 30) occurring in the state of Minnesota from 2009 through 2015 using an ecological exposure to heat waves, and individual demographic data about mothers. Weather data were collected from the Minneapolis–St. Paul airport. The birth records were collected by the Minnesota Department of Health’s Vital Statistics System. Minnesota birth records include information on date of birth, maternal age at child birth, race and ethnicity of the mother, highest grade level completed, and estimated week of gestation [39]. Detailed residence information of mothers was suppressed for confidentiality, but the data includes an indicator of whether the woman resided inside or outside of the seven-county Minneapolis–St. Paul metro area at time of delivery. These anonymous public records were deemed exempt from Institutional Review Board review, but are subject to a data request through the Minnesota Department of Health’s Center for Health Statistics. As of the 2010 census, the population of the state of Minnesota was 5.3 million people. Approximately 60 percent of the population lives in the Minneapolis–St. Paul metropolitan area. Using geospatial analysis tools of census data in ARC GIS and ESRI, we see that 75% of the population of the state of Minnesota lives within zip codes within 75 miles of the Minneapolis–St. Paul airport [40]. To test the assumption that exposure is largely consistent across the state, and that results are consistent regardless of where exposure is measured, 11 additional sites were compared. Agreement of exposure was assessed using several tests of interrater agreement. Additionally, we re-ran regression results using all 12 exposure sites individually. Percent agreement was 0.99 for the 12 sites, with other measures of interrater agreement from 0.51 to 0.99. Additional regression models treating each site as the exposure site showed point estimates of 0.99–1.2 (See Supplementary Figures S1–S3).

The binary outcome of interest is PTB defined as gestational age of less than 37 weeks. We coded this outcome from the “estimated gestation” variable on the birth records. It is typically estimated using recalled date of last menstrual period or ultrasound [41,42,43].

The primary exposure of interest is heat waves. Heat waves have a wide variety of definitions, usually encompassing some combination of heat intensity and heat duration. Smith et al. note that heat waves have been variously measured using standard deviations above a mean temperature, daily maximum temperature, or some measure of heat and humidity at a given duration [44]. In environmental studies, multiple durations of heat waves have been used with lengths ranging from 2 to 7 days [44].

Prior work by Basu et al. and He et al. suggested that a seven-day duration showed the strongest association with PTB, with Basu et al. using an apparent temperature (heat index) and He et al. using dry bulb [13,23]. Building on their findings, we defined a heat wave as a duration and intensity combination of seven days prior to birth with a mean daily high heat index of 37 °C or higher. The heat index is a combined measure of the effect of heat and humidity (see next paragraph for a detailed definition) and is commonly used in humid climates where humidity further reduces the body’s ability to thermoregulate [45]. Overall, our heat wave criterion reflects the 99th percentile of daily high heat index for the time period of this study (June 1–September 30, for the years 2009–2015).

Calculating the heat index and defining heat wave exposures was a multistep process. First, we acquired hourly dry bulb and dew point temperature data collected by the National Weather Service at the Minneapolis–St. Paul airport [46]. Second, to determine the hourly heat index, we combined hourly dew point temperature in degrees Celsius (D_C_) and hourly dry bulb temperature in degrees Celsius (T_C_) using the formula in Equation (1) [47,48]:

Heat Index = −2.719 + 0.994 × T_C_ + 0.016 × (D_C_)^2^ (1)

Third, we recorded the highest heat index observed in a given day. Last, for each day during the study period, we coded as yes or no whether the mean high heat index during the seven days prior equaled or exceeded 37 °C. Because the heat index is a combination of heat and humidity, a heat index of 37 °C can occur across a range of air temperatures (28.5 °C to 35 °C).

Based on the heat wave criterion used here, Minnesota sees anywhere from 0 to 5 heat waves per year. To put this in perspective, nationwide analyses that use a variety of heat wave definitions suggest that Minnesota tends to be fairly representative of heat wave frequencies across the United States. Extreme heat events are most frequently observed in the Southeast and inland Northwest [44].

### 2.2. Covariates

Covariates thought to be potential confounders of the association between heat wave exposure and PTB were extracted from the birth records, and include maternal education, race and Hispanic status (ethnicity), residence status (residing in or out of the seven-county Minneapolis–St. Paul metro area), year of birth, and maternal age. Age has been associated with a u-shaped distribution of PTB—very young women and women over 40 are associated with higher risk of birth complications including preterm [8]. To capture this nonlinear relationship, we created a categorical variable based on maternal age: under 18, 18 to 25, 25 to 40, and over 40.

Race and ethnicity have been correlated with risk of PTB in national studies using 2014–2016 data from the National Vital Statistics System, particularly among non-Hispanic Blacks, who have approximately 50% higher risk of PTB than non-Hispanic Whites [4]. Further, a systematic meta-analysis by Schaaf et al. found highest risk of PTB in births to women who are Black, compared to births to Whites (pooled OR 2.0 95% CI (1.8, 2.2)) [49]. In the same study, births to mothers of Asian ethnicity and Hispanic ethnicity showed no significant association [49]. A 2016 study reports that births to mothers who are American Indians/Alaska Natives have approximately 25% higher risk of PTB [7]. We combined maternal race and Hispanic status into one race/ethnicity categorical variable (White non-Hispanic, Black non-Hispanic, American Indians/Alaska Native non-Hispanic, Asian non-Hispanic, Hispanic, and unknown).

Socioeconomic status has been established as a predictor of PTB and birth weight, and is measured as highest educational attainment [50,51]. We recoded information about the highest year of completed education into three categories of educational attainment: less than high school graduate, high school graduate, and bachelor’s degree or higher. Residence was coded as residing inside or outside of the seven-county metropolitan Minneapolis–St. Paul area. This covariate was included to control for otherwise unknown differences in exposure or standards of care. Year is included as a fixed effect to adjust for potential changes in standard of care.

### 2.3. Statistical Analysis

We constructed six models using all live births for the months of June, July, August, and September for the years 2009–2015. First, we ran a log binomial generalized linear model in Stata (Stata 14, College Station, TX) with PTB as the outcome, heat wave as the primary exposure, adjusting for year of birth via a fixed effect, as shown in Equation (2):

Log(Pr(Preterm)) = B_0_ + B_1_ × Heat Wave + B_2_ × Year (2)

The second model builds on the first model with the addition of covariates to adjust for mother’s age (Age), educational attainment (Education), race/ethnicity (Race_Ethnicity), and residence in the seven-county metro Minneapolis–St. Paul area, shown in Equation (3):

Log(Pr(Preterm)) = B_0_ + B_1_ × Heat Wave + B_2_ × Age + B_3_ × Education + B_4_ × Race_Ethnicity + B_5_ × Metro + B_6_ × Year (3)

Four models tested interactions for Heat Wave × Age, Heat Wave × Education, Heat Wave × Race_Ethnicity, and Heat Wave × Urban. Finally, the effect of Heat Wave × Education × Race was tested using births to Black mothers and White mothers only, with other ethnic groups excluded for simplicity, and to specifically investigate differences in births to Black and White mothers, which show the highest disparity of risk in prior research [49]. Because of small cell counts in some strata (White, <high school), we also repeated this model combining education into two strata—college degree, and less than college degree. We also ran sensitivity analyses using the seven-day average of mean daily heat index and the seven-day average of the daily mean dry bulb temperature to replicate the exposures of Basu et al. and He et al. [13,23], and using seven-day average of each high daily temperature as an additional test of sensitivity. All model coefficients were tested for significance at the 0.05 level, and 95% confidence intervals (CIs) were calculated and reported. All effects are exponentiated and reported as relative risks.

## 3. Results

From 2009 to 2015, a total of 154,157 births occurred in summer months (1 June–30 September) to women in the state of Minnesota (descriptive statistics can be found in Table 1).

Overall, 1.3–1.4% of these women gave birth immediately after experiencing a heat wave. As expected, PTB occurred at a higher percentage in minority races and Hispanics. There was also a higher percentage of PTB in children of women under 18 and women over 40, consistent with earlier studies. The percentage of PTB is similar for women who lived inside the seven-county metropolitan Minneapolis–St. Paul area and women who lived outside of the area. Mothers with high school educational attainment or lower appear to have a higher probability of having a PTB; mothers with a college education appear to have a lower probability.

Regression model results are shown in Table 2.

Based on the first model shown in Equation (1) which included only heat wave exposure and a fixed effect for year, we found that women exposed to a heat wave had 1.14 times higher risk of preterm delivery compared to women with no exposure (CI 1.00–1.30). The higher risk of PTB following exposure to a heat wave remains after further adjustment for race/ethnicity, maternal age, educational attainment, and geographic residence within or outside the metropolitan seven-county area in the second model represented by Equation (2) (1.13 higher risk; CI 0.99–1.3). Adjusted for all other covariates in this model, children born to women who are Non-Hispanic Black have 1.21 times higher risk of PTB compared to children born to women who are Non-Hispanic White (CI 1.15–1.27), American Indian/Alaskan Natives have 1.31 times higher risk (CI 1.18–1.46), and Asians have 1.07 (CI 1.00–1.13) times higher risk. Births to Hispanics have similar risks of PTB as births to Non-Hispanic Whites (CI 0.90–1.02), as do births to women for whom race/ethnicity is unknown (CI 0.67–1.36). All else being equal, women with a high school education have 0.93 times the risk of having a PTB as women failing to obtain a high school education (CI 0.88–0.98), while women with a college degree have 0.79 times the risk (CI 0.74–0.84). Year is not shown as it is not significant in any model, and addition of year to the models does not change the main association of heat waves and PTB. Two-way interactions for Heat Wave × Age, Heat Wave × Race_Ethnicity, Heat Wave × Urban, and Heat Wave × Education were tested but did not improve model fit, and the interaction terms were nonsignificant at alpha = 0.05. The marginal plots of these terms are included as supplementary material—while not significant, there is some suggestion that the effect of heat on preterm birth is elevated for births to women over 40, and reduced for mothers with less than HS education (see Supplementary Figures S4–S7).

Sensitivity analyses of the main effect using alternative estimations of heat waves measured at Minneapolis–St. Paul airport showed similar associations. The relative risk of PTB in births to mothers exposed to a seven-day average of daily mean heat index compared to those not exposed is 1.15 (1.01–1.31). The relative risk of PTB in births to mothers exposed to a seven-day average of daily mean dry bulb temperature compared to those not exposed is 1.14 (0.99–1.30). The relative risk of PTB in births to mothers exposed to a seven-day average of daily high dry bulb temperature compared to those not exposed is 1.06 (0.91–1.23).

**Figure 1:**
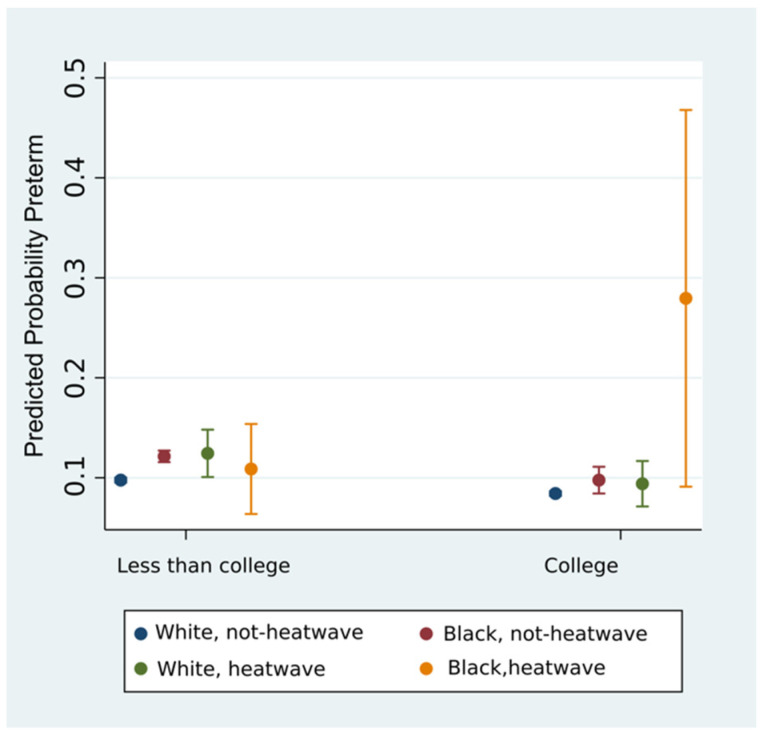
Probability of PTB for White and Black mothers, across strata of education, and exposure to heat waves.

We examined the three-way interaction of Education × Race × Heat Wave using three levels of education (<High School, High School, College) but found that interaction results for White, <HS could not be obtained because so few mothers were White without a high school education. We collapsed education to two strata (college, and less than college) and ran the regression including an interaction term for Education × Race × Heat Wave which revealed that the effect of heat waves on PTB varied across levels of education and race (three-way interaction *p*-value = 0.003). Investigating the marginal effects, we found that 27.94% of college-educated Black women experienced PTB following heat waves, while all other groups including non-college-educated Black mothers and White mothers of any education showed risk of PTB ranging from 8.42 to 12.44% regardless of exposure to heat wave or level of educational attainment. This relationship is shown in Figure 1.

Using post-estimation tools in Stata, we found that births to Black women with college degrees exposed to heat waves show a higher relative risk of PTB compared to births to White women with college degrees in a heat wave (2.97 RR 1.45–6.07 95% CI).

## 4. Discussion

We found a positive correlation between higher risk of PTB in women exposed to heat waves (e.g., seven-day mean high heat index of 37 °C) that did not change after adjustment for maternal age, race/ethnicity, educational attainment, place of residence, and year of birth. In the models discussed here, exposure to a heat wave was associated with 1.13–1.14 times higher risk of a PTB regardless of adjustment for other covariates. These findings are generally consistent with a growing but incomplete body of work demonstrating the association between heat waves and PTB. We do not find any improvement to our model with the addition of interaction terms for heat wave × age, heat wave × urban, heat wave × race/ethnicity, or heat wave × education, but do see a suggestion that the effect of heat waves on preterm birth is exacerbated for births to women over 40. Additionally, we find that the effect of heat wave on the risk of PTB following heat waves is heterogeneous across race and education, as children born to Black mothers with college education show a higher risk of PTB following heat wave than births to all other mothers, regardless of race or heat wave exposure. However, this confidence interval is wide, and while the difference in the point estimates is striking, we cannot rule out overlap across strata of education or race.

Prior studies researching the connection between heat and PTB have had contradictory results. Wang et al. found higher hazard of PTB immediately following exposure to heat waves in research in Australia, particularly later in the term [52]. Similarly, Basu et al. found an 8.6% increase in the risk of PTB in a study of mothers in California exposed to a 5.6 °C increase in temperature immediately prior to birth [13]. Avalos et al. found an 11.6% increase in PTB for each 5.6 °C in increased temperature [22]. Finally, Davand et al. found that a 99th percentile heat index one day prior to birth was associated with a five-day reduction of gestational age [21]. However, other work contradicts these conclusions. A recent study in Montreal found no increased association with heat and PTB [31], and studies in the UK and Germany also found no association between heat and PTB [29,30]. While there is growing support for the association between heat waves and PTB, the science is not settled and there is a variety of exposures and outcome measures.

Our exploration of the differences in the association of heat waves and PTB across different levels of education by race of mothers is the first to consider the effect of heat on the association between race, education, and PTB. Our work builds on prior work in preterm research that found that children born to college-educated mothers who are Black are at a higher risk for small-for-age births [53]. Also, children born to college-educated women who are Black are at higher risk of PTB than those born to higher-educated women who are White, while children born to women who have less than college education have similar levels of PTB regardless of race [11]. Braveman et al. hypothesize that this racial disparity in protection against PTB afforded by education may reflect differences in lifetime accrued stress, through forces associated with racism or different life experiences [11]. These external factors are difficult to capture with standard measures of socioeconomic status such as income or education, as evidenced by a 2005 study that found that even when income levels are equal, individuals who are Black more often live in neighborhoods with higher concentrations of poverty than individuals who are White [54]. Kramer et al. have posited a multifactorial model for differences in PTB outcomes among children of mothers of different race involving long-term stress, preconceptional health, and the environment [55]. Research suggests that neighborhoods with lower measures of socioeconomic status (e.g., neighborhoods with higher poverty or those receiving greater public assistance), often associated with persons of color, have higher exposure to heat due to differing access to parks, higher amounts of paved surface, less residential air conditioning, and less shade [56,57], and historically redlined areas may be as much as 7 degrees Celsius warmer than non-redlined areas [58]. In turn, higher exposure to heat through neighborhood factors might contribute to a higher risk of PTB through decrease in uterine blood flow and increased oxytocin and antidiuretic hormone as discussed in Basu et al. [13]. Another possibility is that any effect of heat may simply add to the sum total of experienced stressors and feed into a multifactorial model framework [11,55]. Overall, our results add to the growing body of work exploring the association between heat waves and PTB, and suggest that environmental stressors may contribute to disparate PTB risk across strata of race and education.

This study is the first of its kind in the state of Minnesota. It has several strengths and a few limitations. The use of heat index allows for use in other regions as it can account for regional variability in humidity and capture the additional effect humidity has on the body’s ability to thermoregulate, where the effects of temperature alone are less where conditions are more arid [45]. The individual-level birth records allow for individual-level covariate measures and examination of interaction terms that aggregated data would not support. The varying effect of environmental exposures is an interesting area of further study. This study would benefit by the inclusion of information on miscarriages or still births as their absence could contribute to biased results. The exposure is ecological, so individuals might have higher or lower true exposure based on, for example, the availability of air conditioning. Similarly, details about specific location of birth would support use of additional weather sites, but as 75% of the state lives within 75 miles of the weather station at Minneapolis–St. Paul airport, and exposure observation at different stations is strongly correlated, we think that it is reasonable to use one site [40]. The exposure is rare in this study group, which may understate the actual population risk in the United States. Additionally, the relative paucity of information may contribute to the wide confidence interval surrounding the relative risk of PTB in college-educated Black women exposed to heat waves. While the data include some useful covariates, additional information such as information about maternal health status, parity, twin status, and insurance information would be useful.

There is opportunity for further research in other regions using additional datasets. While Minnesota can have hot summer days, on average it has short summers and enjoys excellent health care access [59]. Given the ecological nature of the exposure, and national variations in frequency and intensity of heat waves, future research should replicate this work in other regions and include areas where heat shocks are more frequent—this would make the exposure less rare which might help with confidence interval width, and would help to investigate whether the effects of heat waves on PTB are based on some absolute level of heat index, or whether there is some adaptive effect to be gained from living in a more consistently hot environment.

A dataset that includes more information about the births such as birthweight and place of residence would add value to the study, as would information about miscarriages, which are data that could not be captured by this type of data source. In addition to access to more detailed data and data from other places, we are interested in the effect of heat throughout the pregnancy, and possible associations between heat waves in the first or second trimester and PTB or low birth weight are areas of interest. Finally, it would be productive to assess the effect of cold shocks on PTB, especially in this study region, as Minnesota experiences cold winters.

## 5. Conclusions

The occurrence of heat events is expected to increase due to the worsening effects of anthropogenic global warming [60]. This will be seen in both higher mean annual temperatures and in the frequency of heat shocks [37]. In the US, the impacts of climate change on health are expected to have greatest impact on northeastern and midwestern cities [61]. This study demonstrates that even in a relatively cool climate like that of Minneapolis–St. Paul, there is evidence to support the idea that heat waves contribute to higher risk of PTB, and the impact is only likely to increase. While the risk to any one woman is small, the high costs that accompany PTB warrant further research.

The complicated issues of race, education, and heat suggest that a more detailed study of a population with a higher number of people who are Black might be beneficial. While the sample size is small, the magnitude of the effect for the education × race × heat interaction is high, and fits into the growing literature—and suggests an avenue of future work. A clear understanding of the association between heat waves and PTB, along with a better understanding of the unequal way this outcome affects disadvantaged groups, could serve to inform women and caregivers who could plan to minimize exposure, inform policy makers measuring economic impacts of climate change, and reduce the lack of certainty in the relationship between heat waves and PTB. Finally, this work could support further research into the way that climate change disproportionally affects racial and ethnic minority groups, in this case through the complex relationship between heat waves, PTB, and socioeconomic factors like educational attainment and racism.

## Figures and Tables

**Table 1:** Descriptive characteristics of mothers of preterm (<37 weeks) and full-term (≥37 weeks) births, for all live births in Minnesota from 2009 to 2015, and births to women 25 and up from 2009 to 2015.

	*n* = 154,157			
	Gestational Age (Weeks)			
	<37		>37	
Exposure Status Prior Seven Days	*n*	%	*n*	%
Mean heat index < 37 °C	14,542	98.6	137,664	98.8
Mean heat index ≥ 37 °C	212	1.4	1739	1.3
Race/Ethnicity	*n*	%	*n*	%
White, non-Hispanic	10,394	70.45	103,611	74.32
Black, non-Hispanic	1821	12.34	13,334	9.57
AIAN ^a^, non-Hispanic	337	2.28	2273	1.63
Asian, non-Hispanic	1112	7.54	9950	7.14
Hispanic	1063	7.20	9970	7.15
Unknown	27	0.18	265	0.19
Age category	*n*	%	*n*	%
<18	231	1.57	1582	1.13
18 to <25	3158	21.40	28,648	20.55
25 to <40	10,825	73.37	105,657	75.79
≥40	540	3.66	3516	2.52
Residence	*n*	%	*n*	%
Outside seven-county metro MSP ^b^	6297	42.68	60,676	43.53
Inside seven-county metro MSP	8457	57.32	78,727	56.47
Educational attainment (available 2009–2015)	*n*	%	*n*	%
Less than high school education	1782	12.08	13,832	9.92
High school education	7601	51.52	67,806	48.64
College education	5371	36.4	57,765	41.44

Source: Minnesota Center for Health Statistics (MN Annual Natality Statistical File 2009–2015); National Weather Service Automated Surface Observing System (2009–2015). ^a^ AIAN—American Indian, Alaskan Native. ^b^ MSP—Minneapolis–St. Paul seven-county metro area.

**Table 2:** Association of seven-day mean high heat greater than 37 °C with PTB for live births to women in Minnesota from 2009 to 2015, with results for main exposure, and main exposure plus race/ethnicity, mother’s age, metro residence, and mother’s education. All models adjusted for year of birth (fixed effect) and all other covariates.

2009–2015, Births Occurring June 1–September 30		
Summer Births	*n* = 154,157	
	RR ^a^ (95% CI)	Adjusted RR (95% CI)
Mean 7-day heat index ≥ 37 °C ^b^	1.14 (1.00–1.30)	1.13 (0.99–1.28)
Race/Ethnicity ^c^		
Black		1.21 (1.15–1.27)
AIAN		1.31 (1.18–1.46)
Asian		1.07 (1.00–1.13)
Hispanic		0.96 (0.90–1.02)
Unknown		0.95 (0.67–1.36)
Age of mother ^d^		
18 to <25		0.85 (0.74–0.96)
25 to <40		0.86 (0.76–0.98)
≥40		1.22 (1.05–1.41)
Mother’s education ^e^		
High school grad		0.93 (0.88–0.98)
College grad		0.79 (0.74–0.84)
Metro residence ^f^		1.02 (0.99–1.06)
Intercept	0.09 (0.09–0.10)	0.12 (0.11–0.14)

^a^ RR = Relative risk. ^b^ Mean of past 7 days heat index <37 °C is reference. ^c^ Non-Hispanic White is reference race/ethnic. AIAN = American Indian or Alaskan Native. ^d^ Mothers aged less than 18 are reference group. ^e^ Mothers with less than high school education are reference. ^f^ Mothers who reside outside of seven-county metro area of Minneapolis–St. Paul are the reference group. Log binomial models, adjusted for all listed covariates, and fixed effect for year (not shown).

## Supplementary Materials

The following are available online at https://www.mdpi.com/1660-4601/17/17/6391/s1: Figure S1: Sites for comparison of agreement of Heatwaves. Kappa for all sites = 0.53 (*p* = 0.000). Using only the shaded green sites where 85% of the population lives, Kappa = 0.61 (*p* = 0.000). Figure S2: Measures of inter-rater agreement for binary heatwave assignment at 12 sites, where heatwaves are defines as whether, for a day, the prior seven day mean of daily high heat index is ≥the 99th percentile of observations. Figure S3: Independent analyses of main effect using each of the 12 separate sites as the exposure provides effect estimates of 0.99 to 1.20. Figure S4: Probability of preterm birth to mothers ages <18, 18–25, 25–40, >40 for births following heatwaves vs not-heatwaves. Figure S5: Probability of preterm birth to mothers with <HS education, HS diploma, or college education, for births following heatwaves vs not-heatwaves. Figure S6: Probability of preterm birth to mothers who are White, Black, Native, Asian, Hispanic, Unknown for births following heatwaves vs. not-heatwaves. Figure S7: Probability of preterm birth to mothers living in the 7-county area of MSP vs not, for births following heatwaves vs not-heatwaves.
