# Supplementary material for: Association of Summer Heat Waves and the Probability of Preterm Birth in Minnesota: An Exploration of the Intersection of Race and Education
Source: Int J Environ Res Public Health. 2020 Sep 2;17(17):6391. doi: 10.3390/ijerph17176391 (PMC7503599; doi:10.3390/ijerph17176391)
Supplement: Supplementary file 1 [file ijerph-17-06391-s001.docx]

[Figure S1]


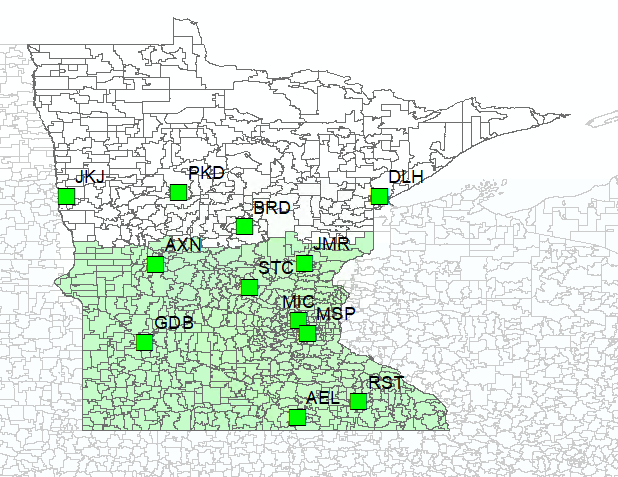


Figure S1: Sites for comparison of agreement of Heatwaves. Kappa for all sites = 0.53 (p=0.000). Using only the shaded green sites where 85% of the population lives, Kappa = 0.61 (p=0.000).

[Figure S2]


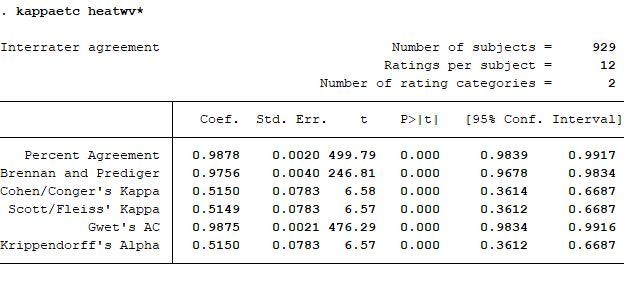


Figure S2: Measures of inter-rater agreement for binary heatwave assignment at 12 sites, where heatwaves are defines as whether, for a day, the prior seven day mean of daily high heat index is >= the 99^th^ percentile of observations.

[Figure S3]


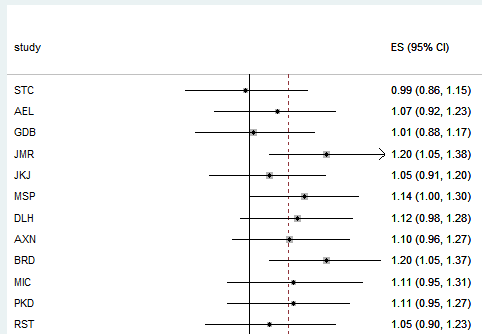


Figure S3: Independent analyses of main effect using each of the 12 separate sites as the exposure provides effect estimates of 0.99 to 1.20.

[Figure S4]


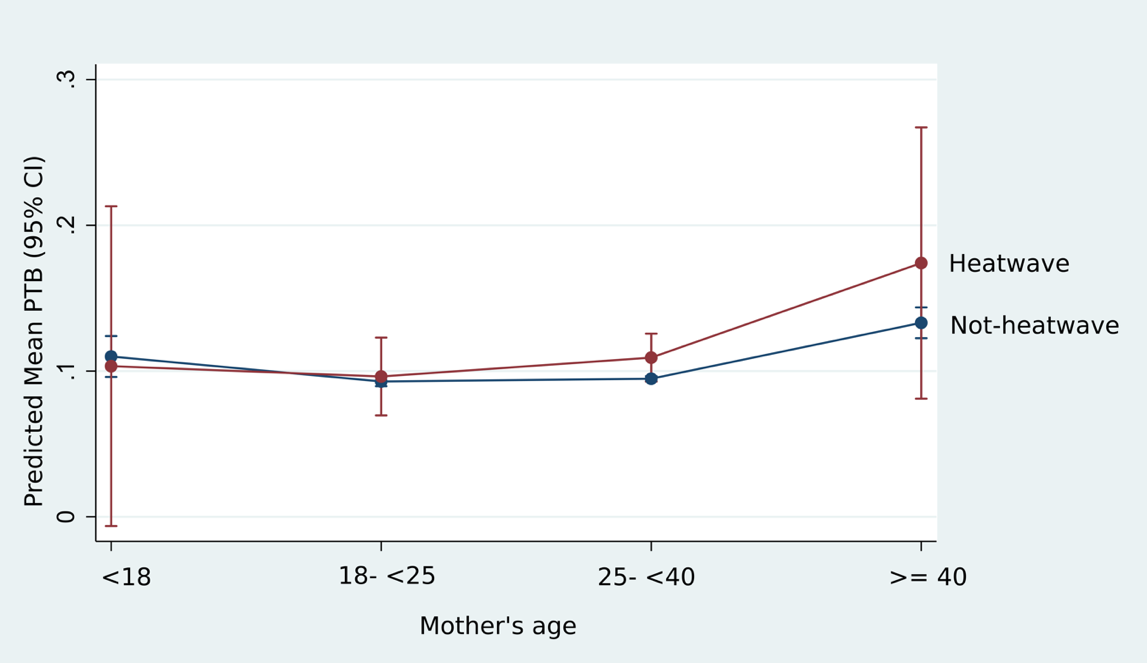


Figure S4: Probability of preterm birth to mothers ages <18, 18-25,25-40,>40 for births following heatwaves vs not – heatwaves.

[Figure S5]


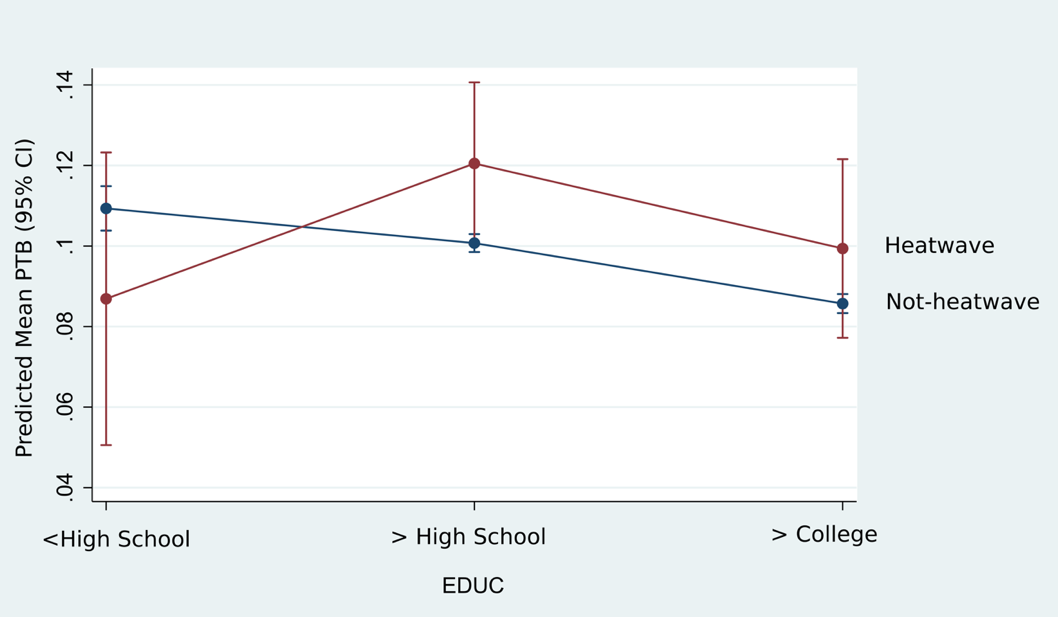


Figure S5: Probability of preterm birth to mothers with <HS education, HS diploma, or college education, for births following heatwaves vs not – heatwaves.

[Figure S6]


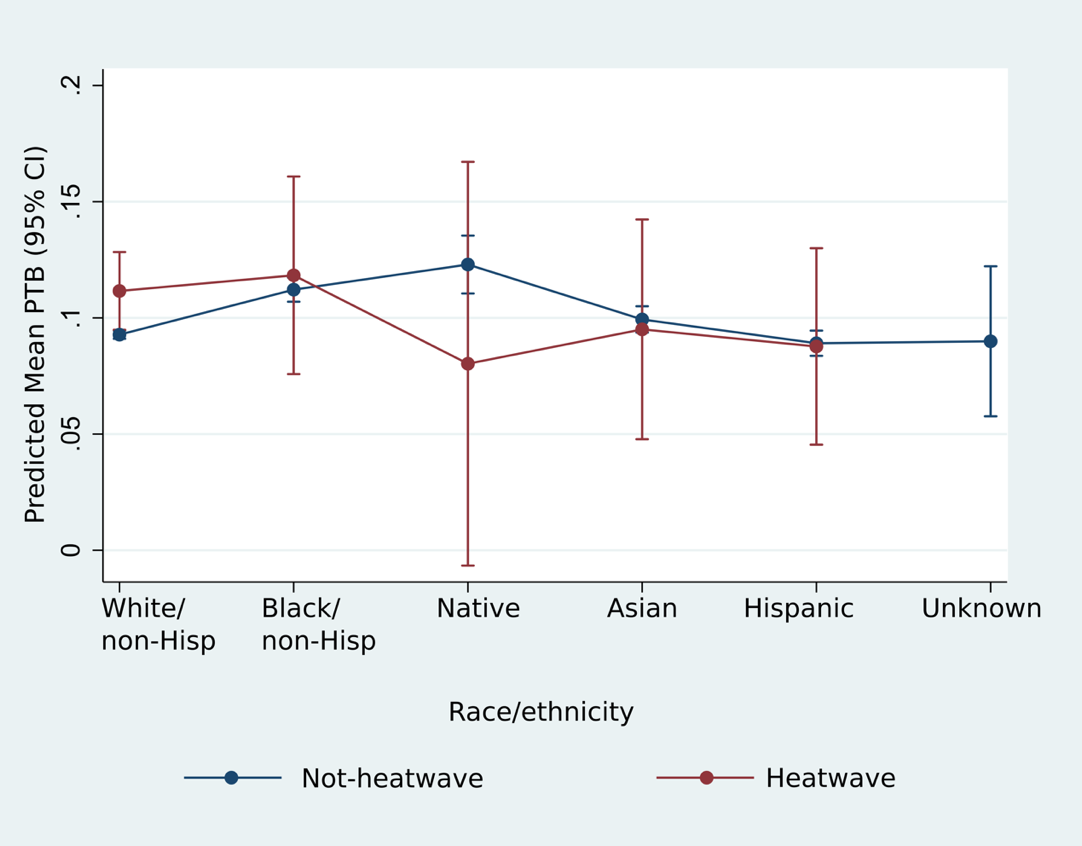


Figure S6: Probability of preterm birth to mothers who are White, Black, Native, Asian, Hispanic, Unknown for births following heatwaves vs not – heatwaves.

[Figure S7]


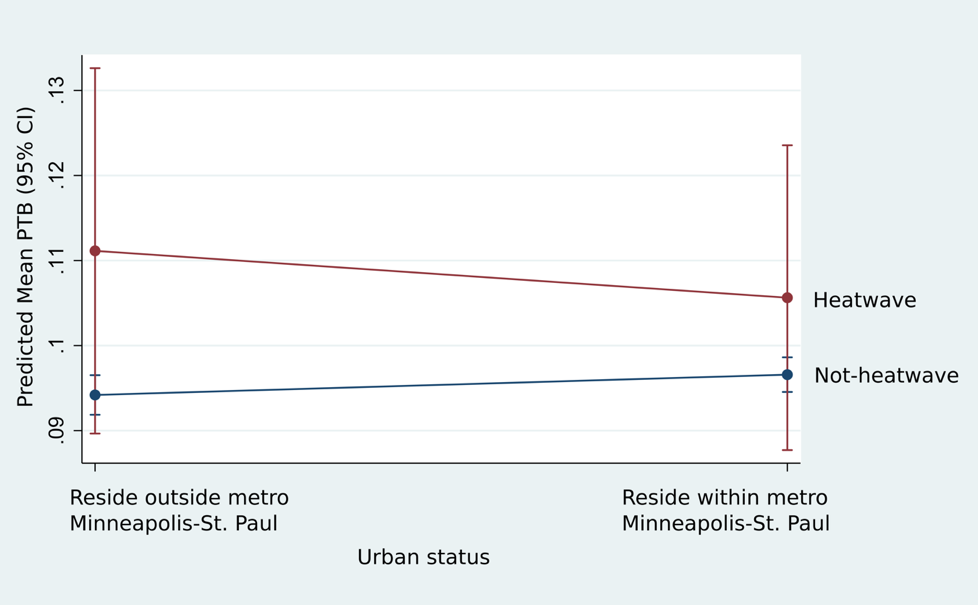


Figure S7: Probability of preterm birth to mothers living in the 7 county area of MSP vs not, for births following heatwaves vs not – heatwaves.
